# Plasmatic exosomes from prostate cancer patients show increased carbonic anhydrase IX expression and activity and low pH

**DOI:** 10.1080/14756366.2019.1697249

**Published:** 2019-12-02

**Authors:** Mariantonia Logozzi, Davide Mizzoni, Clemente Capasso, Sonia Del Prete, Rossella Di Raimo, Mario Falchi, Daniela F. Angelini, Alessandro Sciarra, Martina Maggi, Claudiu T. Supuran, Stefano Fais

**Affiliations:** a Department of Oncology and Molecular Medicine, Istituto Superiore di Sanità, Rome, Italy; b National Research Council, Institute of Biosciences and BioResources, Naples, Italy; c National AIDS Center, Istituto Superiore di Sanità, Rome, Italy; d Neuroimmunology Unit, IRCCS Santa Lucia Foundation, Rome, Italy; e Department of Urology, Policlinico Umberto I, Sapienza University of Rome, Rome, Italy; f NEUROFARBA Department, University of Florence, Section of Pharmaceutical Chemistry, Florence, Italy

**Keywords:** Acidic tumour microenvironment, prostate cancer, carbonic anhydrase IX, plasmatic exosomes, biomarker

## Abstract

Acidity, hypoxia and increased release of exosomes are severe phenotypes of tumours. The regulation of pH in tumours involves the interaction of several proteins, including the carbonic anhydrases which catalyze the formation of bicarbonate and protons from carbon dioxide and water. Among CA isoforms, CA IX is over-expressed in a large number of solid tumours, conferring to cancer cells a survival advantage in hypoxic and acidic microenvironment, but there isn’t evidence that CA IX expression could have a real clinical impact. Therefore, in this study for the first time the expression and activity of CA IX have been investigated in the plasmatic exosomes obtained from patients with prostate carcinoma (PCa). For this purpose, the study was performed through different methodological approaches, such as NTA, western blot analysis, enzyme activity assay, Nanoscale flow cytometry, ELISA, confocal microscopy. The results showed that PCa exosomes significantly overexpressed CA IX levels and related activity as compared to healthy donors. Furthermore, CA IX expression and activity were correlated to the exosome intraluminal pH, demonstrating for the first time that PCa exosomes are acidic. Our data suggest the possible use of the exosomal CA IX expression and activity as a biomarker of cancer progression in PCa.

## 1. Introduction

Extracellular acidity is a common phenotype of malignant tumours due to the “Warburg effect”. As a consequence, the anaerobic metabolism of glucose, which is triggered by hypoxia, leads to a massive accumulation of lactic acid and H^+^ within the cytoplasm and in the extracellular microenvironment^1–6^. Moreover, the extracellular acidity induces a selective pressure leading to the clonal selection of cancer cells that can survive in such a hostile condition^7^. Cancer cells survive and proliferate thanks to a series of innate mechanism conceivably common to all malignant tumours^8–10^. Among these, the proton pumps play a crucial role, reducing intracellular pH and acidifying the extracellular environment^9^^,^^11^^,^^12^. Therefore, tumour cells up-regulate proton exchangers and transporters throwing out excess protons through Vacuolar ATPase (V-ATPase), Na+/H + exchanger (NHE), monocarboxylate transporters (MCTs), cotransporter sodium bicarbonate (NBC), and carbonic anhydrase 9 (CA IX)^2^^,^^11–13^. Chemoresistance of cancers and the increase in exosome release are two well-known effects of the extracellular acidification^14–18^. Exosomes are extracellular nanovesicles of 40–180 nm secreted by all cells and capable of modulating physiological or pathological processes, including tumour progression, through transmitting into target cells their cargo (lipids, proteins, DNA, mRNA or microRNA)^18–24^. Exosomes are involved in a broad panel of cellular phenomena, including intercellular communication, elimination of toxic substances and drug delivery^14^^,^^18^^,^^25^^,^^26^. The acid release of the exosomes is key in tumour growth, tumour progression and metastasis^18^^,^^24^^,^^27^. High plasmatic levels of extracellular vesicles are related with the tumour mass^28^^,^^29^. It has been demonstrated that, independently from the tumour histotype, the tumour cell lines cultured at pH 6.5 released significantly higher levels of exosomes as compared to the same cells cultured at physiological pH (7.4)^16^^,^^17^. Recently, it has been shown that the microenvironmental low pH was consistent with a change in exosome cargo as well as with some tumour biomarkers such as Prostate Specific Antigen (PSA) and carbonic anhydrase (CA) IX^16^^,^^30^. CA IX is a zinc metalloenzyme belonging to a broader group of 15 isoforms involved in the catalysis of carbon dioxide hydration to bicarbonate and proton. Among these isoforms, CA IX has high clinical relevance in cancer therapy since it is implicated in tumorigenesis^13^^,^^31^^,^^32^ and considered a marker of tumour hypoxia^33–36^. CA IX is overexpressed in a large number of solid tumours, whereas its expression in normal tissues is quite limited, suggesting its role as valuable tumour biomarker. Furthermore, CA IX has been validated as a target of new therapies against hypoxic tumours with one sulphonamide inhibitor (SLC-0111) in Phase Ib/II clinical trials^13^^,^^30^^,^^37–39^^(p20)^^,40–42^. Indeed CA IX is the most widely expressed gene in response to hypoxia, playing a pivotal role in tumour pH regulation^13^^,^^30^^,^^37^^,^^41^; thus conferring to cancer cells a survival advantage in hypoxic and acidic microenvironments^43^. Thus, it is clear that the acidic microenvironment induces the up-regulation of the CA IX expression and activity in both tumour cells^30^^,^^44^ and tumour released exosomes^30^. However, the possibility that CA IX expression and activity in cancer cell-derived exosomes could represent a new valuable tumour marker has not yet been tested in clinical samples.

In the present study, the expression and the activity of CA IX were detected in the plasmatic exosomes obtained from patients affected and non-affected by prostate carcinoma (PCa). Moreover, CA IX behaviour was correlated to the intraluminal pH of plasmatic exosomes in both patients and controls. Our results indicate that exosomal CA IX showed a higher expression and activity when compared to the controls, which were consistent with a lower intraluminal pH of the plasmatic exosomes of PCa. These data suggest that exosomal CA IX might be an interesting biomarker of cancer progression.

## 2. Materials and methods

### 2.1. Human plasma samples

Human plasma samples were collected from EDTA-treated whole blood, 5 ml into BD Vacutainer^®^ K3-EDTA-coated collection tubes (Beckton Dickinson, USA), from prostate cancer (PCa) patients (*n* = 8) and healthy donors used as controls (CTR) (*n* = 8) attending to department of Urological Sciences, Policlinico Umberto I, Sapienza University of Rome, Italy. The study was approved by the ethics committee of IstitutoSuperiore di Sanità (ISS, Rome, Italy) on 18/04/2017 (Rif. Prot. PRE-275/17). The study was conducted in accordance with the current International Conference on Harmonisation guidelines for Good Clinical Practice and the principles of the Declaration of Helsinki. All the participants provided written informed consent.

The study population included the prostate cancer group (PCa) and the healthy donors group (CTR). The PCa group consisted of 8 male individuals consecutively referred to department of Urological Sciences, aged from 45 to 75 years, with a histologically confirmed diagnosis of prostate adenocarcinoma (prostate biopsy). None of cases were submitted to androgen deprivation therapies or other therapies that can influence PSA determination. All cases were stratified in risk classes (EAU classification) on the basis of total PSA levels (>4.5 ng/ml), Gleason score and clinical stage.

The control group (CTR) consisted of 8 male individuals consecutively referred to Urological Sciences Department with the following inclusion criteria: age from 18 to 39 years; no clinical evidence of BPH or PCa (digital rectal examination (DRE) and ultrasonography US); prostate volume less than 30 cc; no familiarity for PCa; no therapies that can influence PSA determination.

### 2.2. Preparation of exosomes from plasma of patients and healthy donors

To obtain exosomes from plasma samples, EDTA-treated blood from 8 patients with prostate cancer (PCa) and 8 healthy donors (CTR) was centrifuged at 400 g for 20 min. Plasma was then collected and stored at −80 °C until they will be analysed for the expression of CA IX. Upon thawing, 1 ml of plasma samples was subjected to the same centrifugal procedure as previously described^45^^,^^46^ in order to pellet exosomes. Plasma samples were centrifuged for 1 h 30 min at 110 000 g using a Fiberlite™ F50L-24 × 1.5 Fixed-Angle Rotor, K-Factor: 33 (ThermoFisher Scientific, USA) in Sorvall WX Ultracentrifuge Series (ThermoFisher Scientific, USA).

### 2.3. Nanoparticle tracking analysis

Nanoparticle tracking analysis (NTA) from Malvern (NanoSight NS300, Malvern Instruments, Malvern, UK) was used for size distribution and concentration measurements of exosomes samples in liquid suspension from the properties of both light scattering and Brownian motion. The NanoSight NS300 with a 405-nm laser instrument (Malvern Instruments, Malvern, UK) was used to detect nanovesicles. Five videos of typically 60-s duration were taken. Data were analysed using the NTA 3.0 software (Malvern Instruments) which was optimised to first identify and then track each particle on a frame-by-frame basis. The Brownian motion of each particle was tracked using the Stokes–Einstein equation: D° = kT/6π*η*r, where D° is the diffusion coefficient, kT/6π*η*r = f_0_ is the frictional coefficient of the particle, for the special case of a spherical particle of radius r moving with uniform velocity in a continuous fluid of viscosity *η*, k is Boltzmann’s constant, and T is the absolute temperature.

### 2.4. Western blot analysis

To perform Western blot analysis, the plasmatic exosomes pellet was resuspended in 1 ml of PBS and it was further purified by using 30% sucrose in deuterium oxide (D_2_O, ACROS Organics, fisher scientific, USA) density gradient ultracentrifugation for 18 h at 110,000 *g*, in order to eliminate contaminants^46^^,^^47^. Density gradient ultracentrifugation was performed by using TH-641 Rotor (ThermoFisher Scientific, USA). The 12 fractions obtained were washed in PBS for 1 h at 110,000 *g* and then were resuspended in CHAPS buffer 1x for subsequent experimental analysis.

Lysates were prepared in CHAPS buffer (10 mM Tris-HCl [pH 7.4], MgCl_2_ 1 mM, EGTA 1 mM, CHAPS 0.5%, glycerol 10%, β-mercaptoethanol 5 mM, PMSF 1 mM) containing protease inhibitor cocktail. Exosomes lysates were subjected to electrophoresis on SDS polyacrylamide gels and transferred to nitrocellulose membranes (ProtranWhatman, Dassel, Germany). After blocking in 5% dry milk in PBS 1X, membranes were hybridised with primary antibodies: M75^48^, anti-CD81 (B-11, Santa Cruz Biotechnology, USA), anti-Alix (3A9, ThermoFisher Scientific, Waltham, MA, USA) mouse monoclonal antibodies. After incubation with appropriate peroxidase-conjugated anti-IgG (AmershamBiosciences, Milan, Italy), membranes were revealed using the ECL Chemiluminescent Substrate, (ThermoFisher Scientific, USA).

### 2.5. ELISA for CA IX

96 well-plates (Nunc, Milan, Italy) were coated with 4 µg/ml rabbit polyclonal anti-CD81 antibody (clone PA5-79003, Thermo Fisher Scientific, USA) in 100 µl/well of PBS and incubated overnight at 4 °C. After 3 washes with PBS, 100 µl/well of blocking solution (PBS containing 0.5% BSA) were added at room temperature for 1 h. Following 3 washes in PBS, exosomes purified from 1 ml of plasma were suspended in a final volume of 50 µl and incubated overnight at 37 °C. After 3 washes with PBS, M75 mouse monoclonal antibody^48^ was added to each well and incubated for 1 h at 37 °C. After 3 washes with PBS, anti-mouse HRP-conjugated was incubated in each well for 1 h at RT. After the final 3 washes with PBS, the reaction was developed with Blue POD for 15 min (Roche Applied Science, Milan), and blocked with 4 N H_2_SO_4_ stop solution. Optical densities were recorded at 450 nm.

### 2.6. Enzyme activity of CA IX

Exosomes were obtained from plasma of 8 prostate cancer patients (PCa) and 8 healthy donors (CTR). Exosome extracts were prepared at 4 °C using the lysis buffer (CHAPS buffer 1x) containing 1% Triton X-100, 10mMTris-HCl (pH 7.4), MgCl_2_ 1 mM, EGTA 1 mM, CHAPS 0.5%, glycerol 10%, β-mercaptoethanol 5 mM, and supplemented with a cocktail of protease inhibitors. Aliquots of exosomes extracts containing 1 µg of total protein were used to determining the hydratase activity. The enzymatic assay was performed at 0 °C using CO_2_ as substrate following the pH variation due to the catalysed conversion of CO_2_ to bicarbonate. Bromothymol blue was used as the indicator of pH variation. The production of hydrogen ions during the CO_2_ hydration reaction lowers the pH of the solution until the colour transition point of the dye is reached. The time required for the colour change is inversely related to the quantity and activity of CAs present in the sample. Wilbur–Anderson units were calculated according to the following definition: One Wilbur–Anderson unit (WAU) of activity is defined as (T0 − T)/T, where T0 (uncatalyzed reaction) and T (catalysed reaction) are recorded as the time (in seconds) required for the pH to drop from 8.3 to the transition point of the dye (pH 6.8) in a control buffer and in the presence of enzyme, respectively. Enzyme activity was expressed as CA activity/mg of total protein. Protein concentration was determined using the Bio-Rad protein assay.

### 2.7. Flow cytometry analysis of exosomesfor evaluation of exosomal pH

Exosomal pH was evaluated by Nanoscale Flow Cytometry using the pH-sensitive fluorescent probe BCECF AM (2',7'-Bis-(2-Carboxyethyl)-5-(and-6)-Carboxyfluorescein, Acetoxymethyl Ester) (B-1170, Molecular Probes, Invitrogen, ThermoFisher Scientific, USA). Exosomes purified from 1 ml of 8 PCa and 8 CTR plasma samples were diluted in PBS in a final volume of 40 µl. Anti-human CD81 allophycocyanin (APC) conjugated (Beckman Coulter; Brea, CA, USA) and BCECF AM (B-1170, Molecular Probes, Invitrogen, ThermoFisher Scientific, USA) were added to the exosome preparation at optimal pre-titered concentrations and left for 20 min at RT. Anti IgG2a APC (Beckman Coulter; Brea, CA, USA) was used for isotype control.500 µl of PBS were added to samples before the acquisition on the CytoFLEX flow cytometer (Beckman Coulter, Brea, CA, USA). The cytometer was calibrated using a mixture of non-fluorescent silica beads and fluorescent (green) latex beads with sizes ranging from 110 nm to 1300 nm. This calibration step enables the determination of the sensitivity and resolution of the flow cytometer (fluorescent latex beads) and the size of extracellular vesicles (silica beads). CD81 was labelled in allophycocyanin (APC) that absorbs and emits red light (650 and 660 nm max, respectively). BCECF, which emits fluorescence once it enters into the acidic milieu, absorbs and emits green light (488 and 525/640 nm). All samples were acquired at low flow rate for the same amount of time in order to obtain an estimate of absolute counts of exosomes comparable between various samples. The analysis of the data was performed with FlowJo software (FlowJo, LLC; Ashland, Oregon, USA)^16^.

### 2.8. Cell line

Human prostate carcinoma cell line (LNCaP) is derived from a metastatic site (left supraclavicular lymph node) of a 50-year-old Caucasian male (blood type B+) with confirmed diagnosis of metastatic prostate carcinoma (IstitutodeiTumori di Milano). Tumour cells were negative for Mycoplasma contamination as routinely tested by PCR (Venor^®^GeM, Minerva Biolabs, Germany). The cells were maintained in RPMI 1640 without sodium bicarbonate culture medium at pH 6.5 supplemented with antibiotics and 10% foetal calf serum (FCS) (Invitrogen, Milan, Italy), at 37 °C in humidified 5% CO_2_. The acid cell culture medium (pH 6.5) was obtained by the addition of 1 M HCl solution. The pH was measured with a pH 123 Microprocessor pH Metre (Hanna Instruments, Milan, Italy). LNCaP cells were slowly adjusted to pH 6.5 for a sufficient time starting from unbuffered conditions, allowing tumour cells to acidify the microenvironment themselves. After five days in unbuffered medium, measured pH of the culture was 6.5. Thus, we progressively conditioned the pH of the cultures starting from 7.4 until pH 6.5 in a time ranging from three to four weeks, allowing the cells to not be exposed to short-term pH stress^17^.

### 2.9. Laser scanning confocal microscopy (LSCM)

About 1 x 10^5^ cells/well of LNCaP cells cultured at pH 6.5 were seeded on coverslips in 24-well plate. After 24 h, cells were stained with BCECF AM (Molecular Probes, ThermoFisher Scientific, USA) (10 µM) for 30 min at 37 °C. After being fixed in 4% paraformaledehyde, cells were blocked for 1 h at RT in PBS with 1% BSA and labelled with CD9-PE mouse monoclonal antibody (clone LM-L13 (RUO), BD Biosciences, USA) for 1 h at RT; then cells were incubated with M75 mouse monoclonal antibody^48^ for 1 h at RT, following from incubation with anti-mouse Alexa Fluor^®^ 647 secondary antibody (Abcam, UK). After washes in PBS, coverslips with DAPI + ProLong (Vector Laboratories, Burlingame, CA) were transferred on microscope slides and images were acquired with an inverted microscope (Nikon Ti-E)equipped with a confocal spectral imaging system (Nikon D Eclipse C1si) using a (Nikon) PlanApo objective 60× oil (numerical aperture 1.4). Excitation light was obtained by a Laser Dapi (408 nm) for DAPI, an Argon Ion Laser (488 nm) for BCECF, Diode Laser HeNe (561 nm) for CD9-PE, and a Red Diode Laser (638 nm) for Alexa 647. Emitted fluorescence was recorded in spectral – frame lambda mode. DAPI emission was recorded from 415 to 485 nm, BCECF emission was recorded from 495 to 550 nm, CD9-PE emission was recorded from 583 to 628 nm, and Alexa 647 from 634 to 750 nm. Images recorded have an optical thickness of 0.20 µm and have been analysed by the C1-LCSI EZ-C1 software for spectral analysis. Signals from different fluorescent probes were taken in sequential scanning mode, several fields were analysed for each labelling condition, and representative results are shown.

### 2.10. Statistical analysis

Results in the text are expressed as means ± standard error (SE), calculated using the GraphPad Prism software. The statistical analysis was done with an unpaired *t*-test (Student’s *t*-test).

## 3. Results

### 3.1. CA IX expression is up-regulated in exosomes isolated from plasma of prostate cancer (PCa) patients

**Figure 1.**
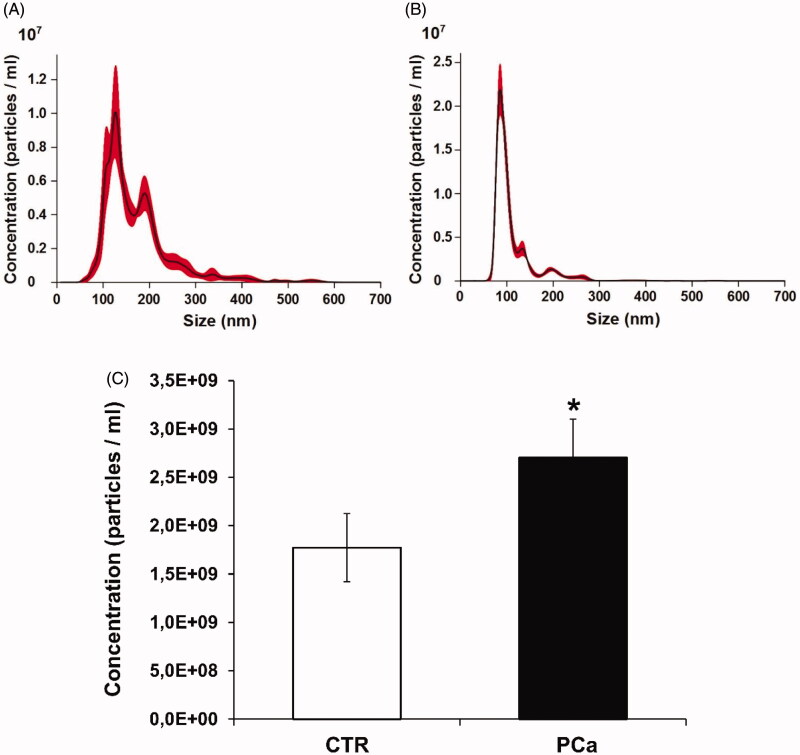
Nanoparticle tracking analysis (NTA) quantification of exosomes released from plasma of PCa patients and CTR. (A) NTA distribution of CTR exosomes. (B) NTA distribution of PCa exosomes. (C) Concentration (particles/ml) of exosomes. Mean ± SE of plasma exosomes from 8 CTR and 8 PCa patients are shown. The *p* values was <.1 in PCa plasma respect to CTR plasma exosomes. *p < .1.

**Figure 2.**
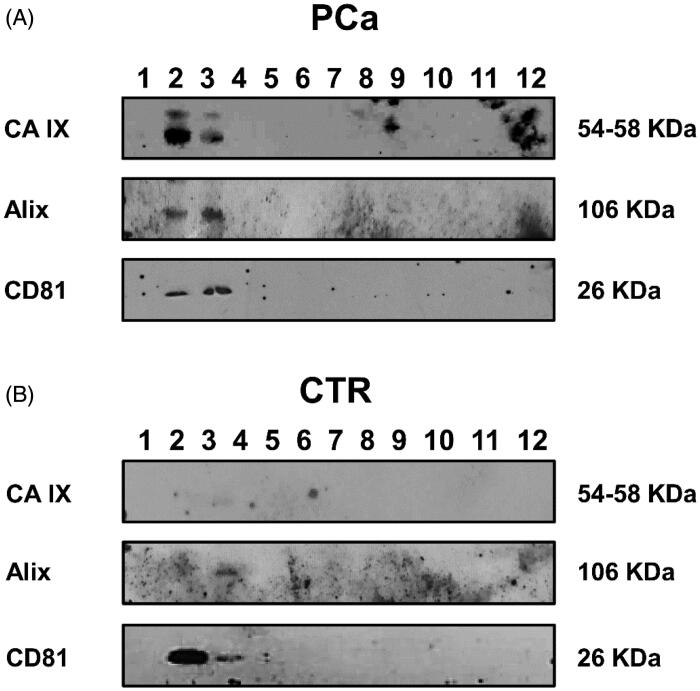
Western blot analysis of plasmatic exosomes from PCa patients and CTR after 30% sucrose density gradient ultracentrifugation for housekeeping markers (Alix and CD81) and CA IX expression. (A) Protein characterisation of exosomal fractions purified from plasma of PCa patients performed with anti-Alix, anti-CD81 and M75 (CA IX). (B) Protein characterisation of exosomal fractions purified from plasma of CTR performed with anti-Alix, anti-CD81 and M75 (CA IX).

A series of experiments aimed at evaluating the level of CA IX expression and activity in exosomes purified from PCa and control (CTR) plasma has been performed. Exosomes isolated by ultracentrifugation were characterised and quantified by Nanoparticle Tracking Analysis (NTA) and the expression of the exosomal markers Alix and CD81. PCa patients showed higher plasmatic levels of exosomes than the controls (*p* < .1) (Figure 1(A–C)). Moreover, exosomes from PCa patients were more homogeneous in size distributionin respect to plasmatic exosomes from CTR (Figure 1(A–C)). Thus, to eliminate contaminants, the Western Blot Analysis of plasmatic exosomes purified by differential ultracentrifugation and further using 30% sucrose density gradient ultracentrifugation was performed^46^^,^^47^. The resulting fractions were blotted for CA IX and exosomal markers (Alix and CD81). The results showed that CA IX expression was up-regulated in exosomal purification lysates from PCa plasma patients (Figure 2(A)) as compared to the exosomal fractions of CTR plasma (Figure 2(B)). Figure 2 also shows that the CA IX band corresponded to the typical exosome fractions as identified by the expression of Alix and CD81 in the second and third fractions of the density gradient. As shown in Figure 2(A), CA IX in PCa exosomes is evidenced by the presence of two bands (58/54 kDa). The CTR exosomes (Figure 2(B)) exclusively showed the Alix and CD81 bands.

### 3.2. CA IX positive exosomes are over-expressed in PCa plasma patients: characterisation and quantification by ELISA test

**Figure 3.**
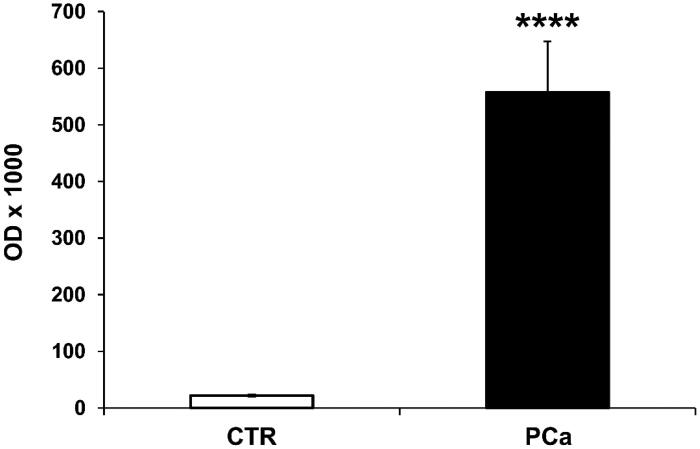
Detection, quantification and characterisation of CAIX+/CD81+ exosomes purified from PCa and CTR plasma by ELISA test. ELISA analysis of CAIX and CD81 expression in exosomes purified from plasma of 8 PCa patients and 8 CTR (1 ml for each sample). Rabbit polyclonal anti-CD81 antibody was used for the capture of exosomes on the plate. Expression levels of exosomal CAIX were expressed as means ± ES. The *p* values was <.0001 in PCa plasma exosomes respect to CTR plasma exosomes. ****p < .0001.

To support this set of results, we exploited an immunocapture-based ELISA assay to quantify and characterise CA IX expression levels in exosomes purified from 1 ml of either PCa or CTR plasma, by seeding the same amount of exosomes preparations (50 µl). The results showed that the CA IX positive exosomes were 25-fold higher in plasma of PCa patients (558 ± 90) than in CTR (22 ± 2), (*p* < .0001) (Figure 3). The plasma deprived of exosomes was entirely negative for both markers (data not shown).

### 3.3. CA enzymatic activity analysis of PCa and CTR plasma exosomes

**Figure 4.**
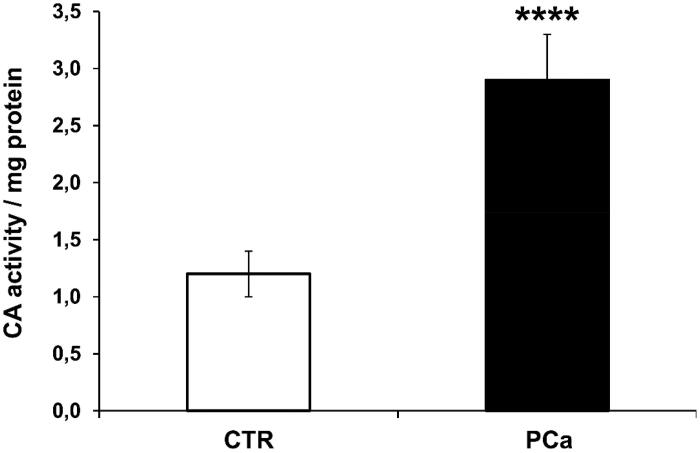
Analysis of CAIX enzymatic activity of plasma exosomes from PCa and CTR. CA IX activity is shown for plasma exosomes purified from 8 PCa patients and 8 CTR. The results were expressed as means ± SD of CA-activity/mg protein found in exosomes. The *p* values was <.0001 in PCa plasma exosomes respect to CTR plasma exosomes. ****p < .0001.

The CA IX-activity of exosomes isolated from plasma of PCa and CTR has been analysed using the colorimetric CA assay as described in the “Materials and Methods” section. The results showed that the CA-activity/mg protein found in exosomes isolated from PCa plasma (2.9 ± 0.4) was 2.4-fold higher as compared to exosomes purified from CTR plasma (1.2 ± 0.2) (*p* < .0001) (Figure 4); thus supporting that the increased CA IX expression in plasmatic exosomes of PCa patients was consistent with a real enzyme activity up-regulated in PCa plasma exosomes. This result is, of course, of paramount importance since circulating exosomes may export to other tissues a function that appears to be associated with malignancy.

### 3.4. Exosomes from PCa plasma patients are acidic

Recently, our groups showed that the tumour acidic microenvironment markedly increases the number of exosomes, also those expressing CA IX^16^^,^^17^^,^^30^. However, never the acidity of the tumour microenvironment has been associated with the intraluminal acidification of the exosome released by cancer cells. Thus, in this set of experiments we studied the intraluminal pH in plasmatic exosome preparations from either PCa or CTR. Plasmatic exosomes purified from PCa and CTR samples were analysed using Nanoscale Flow Cytometry (Cytoflex) for the presence of typical exosomal CD81 marker, and the expression of the fluorescent indicator for cytosolic pH, BCECF.

**Figure 5.**
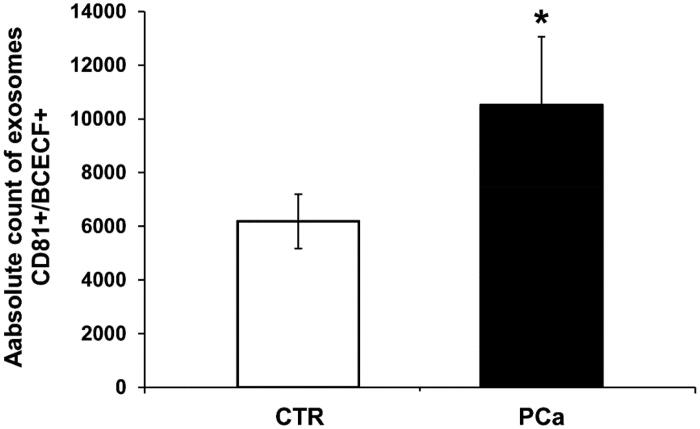
Nanoscale flow cytometry of plasma exosomes in PCa and CTR for intraluminal pH evaluation. The cytometer was calibrated using a mixture of non-fluorescent silica beads and fluorescent (green) latex beads with sizes from 110 nm to 1300 nm. The exosome preparation derived from plasma of 8 PCa patients and 8 CTR were stained 20 min at RT with anti-CD81 antibody and BCECF AM (10 µM) and analysed using flow cytometry. The double-positive events were then analysed for their size, based on the calibration with beads. Cumulative data are shown of the absolute number of CD81+/BCECF + exosomes of size less than 180 nm recovered from the plasma samples. Data are expressed as means ± SE. The *p* values was <.1 in PCa plasma exosomes compared to CTR. *p < .1.

Double-positive events were counted and analysed by size. The results showed that the number of double-positive exosomes smaller than 180 nm was higher in PCa patients as compared to CTR (Figure 5). Figure 5 shows the absolute average number of CD81+/BCECF + exosomes (sizes less than 180 nm) obtained from either PCa or CTR plasma. In particular, the amount of acidic exosome was 2-folds higher in PCa plasma (10 510 ± 2551) as compared to CTR plasma (6184 ± 1015) (*p* < .1). These results evidenced for the first time that an intraluminal acidic pH characterised the exosomes of PCa plasma patients. It is consistent with the higher expression and activity of a CA IX, a protein associated with both acidity and hypoxia. The statistical analysis was performed using the unpaired *t*-test with the GraphPad Prism 6 software.

### 3.5. Acidic tumour microenvironment up-regulates CA-IX expression

**Figure 6.**
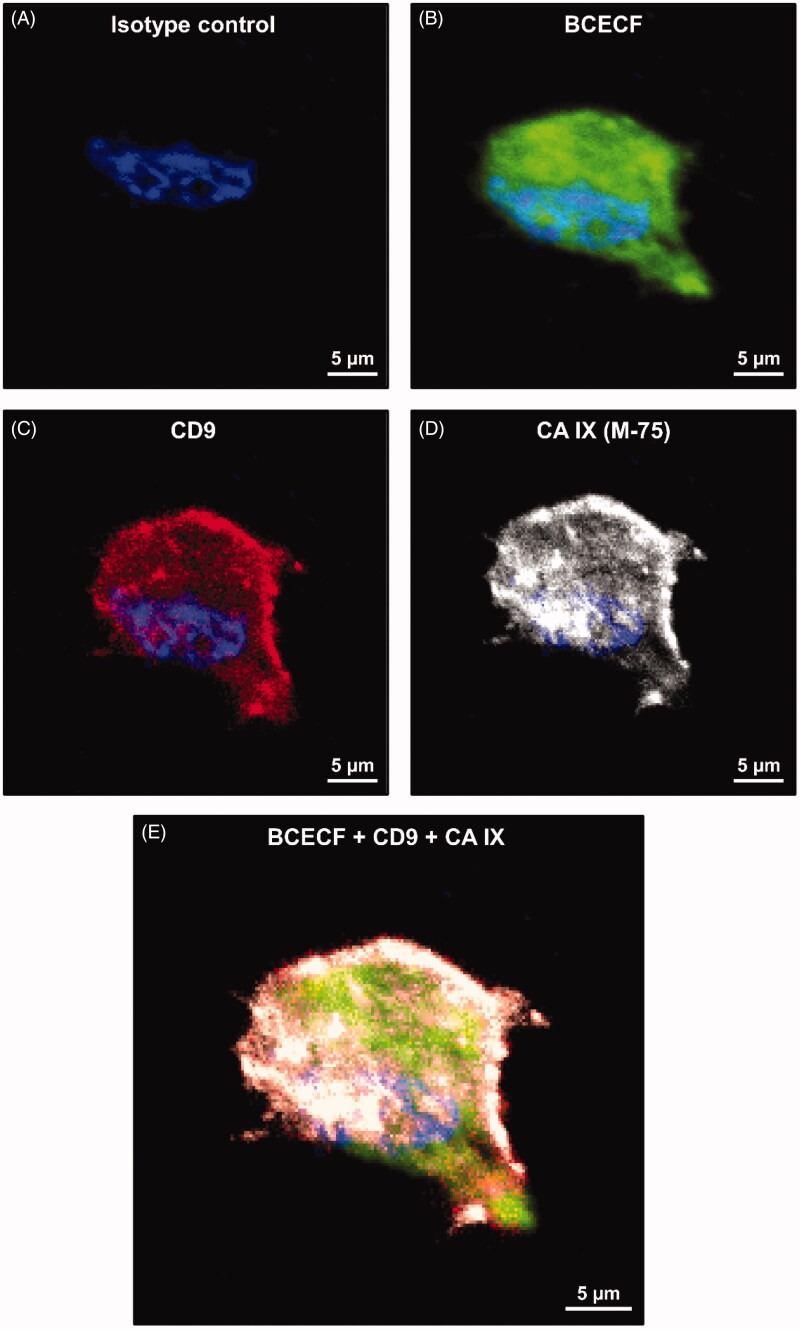
Confocal microscopy analysis of BCECF and CA IX expression in LNCaP cell line cultured at pH 6.5. (A) Isotype control cell. (B) LNCaP cell labelled with BCECF (green signal). (C) LNCaP cell labelled with CD9 monoclonal antibody (M-L13, RUO, red signal). (D) LNCaP cell incubated with M75 and then with Alexa Fluor^®^ 647 secondary antibody for CA IX detection (gray signal). (E) Merged image of different signals on LNCaP cells: BCECF (green signal), CD9 (red signal), CA IX (grey signal), nucleus (blue signal of DAPI).

The CA IX expression was determined and compared to the level of intracellular acidity in a human prostate cancer cell line (LNCaP) cultured at pH 6.5. Intracellular acidity was analysed by Confocal Microscopy using BCECF, the fluorescent tracer. The results showed a massive cytoplasmic distribution of BCECF (in green) (Figure 6(B)) in LNCaP cultured in acidic conditions. This is due to the intake of BCECF into the cells triggered by the low intracellular pH. The BCECF staining was consistent with the CD9 staining (an exosome marker), predominantly at the plasma membrane level, while detectable at the cytoplasm level as well (Figure 6(C)). Interestingly, we showed a similar distribution of CA IX expression (in gray). It is mainly localised at the plasma membrane, also into the cytoplasm, but associated with the nuclear membrane as well (Figure 6(D)). Figure 6(E) shows the merged distribution within the LNCaP cells of the four fluorescent dyes used to identify the nucleus (blue), BCECF (green), CD9 (red) and CA IX (gray). CD9/CA IX is co-localised within low pH cultured cancer cells. The staining partially overlaps the BCECF fluorescence suggesting that CA IX is expressed during exosome release in acidic conditions and that these exosomes are acidic as well.

## 4. Discussion

Among the phenotypes common to virtually all malignant tumours are hypoxia, acidity, and low nutrient supply^1–5^ and ROS hyperproduction, as recently described^49^. In particular, acidic extracellular microenvironment induces a selective pressure leading to selection of cancer cells armed to survive in extreme microenvironmental conditions^7^. Between these adaptive advantages, cancer cells implement a series of strategies, making them very similar to unicellular organisms^8^^,^^10^. However, to prevent intracellular H^+^ accumulation cancer cells upregulates proton exchangers that extrude protons in excess contributing to acidify extracellular tumour microenvironment^9^^,^^11^^,^^12^. Among these proton exchangers, a primary role is played by CA IX, an enzyme overexpressed in many types of cancers, including prostate cancer, with the potential to represent both a promising tumour biomarker and a specific target for future cancer therapies^13^^,^^30–33^^,^^37–41^^,^^44^. Under the pressure of the acidic microenvironment, CA IX expression and activity are upregulated in both tumour cells^30^^,^^44^ and the exosomes released extracellularly^30^. In order to support a real clinical impact of the previous information, we performed a pilot clinical study in which exosomes purified from plasma of prostate cancer patients have been compared to plasmatic exosomes from healthy controls. We analysed the expression and function of CA IX in plasmatic samples, hypothesising that this was related to the decreased intraluminal pH. The results of this study show for the first time that (i) exosomes purified from plasma of patients with prostate cancer express significantly higher levels of CA IX, through different methodological approaches, such as western blot analysis, in both the whole exosome lysates and sucrose gradients, and immunocapture based ELISA; (ii) the increased CA IX exosome expression corresponded to a real function inasmuch as exosomes purified from plasma of patients with prostate cancer showed a significantly higher CA-activity; (iii) exosomes purified from plasma of patients with prostate cancer are acidic, as shown by Nanoscale Flow Cytometry and (IV) CA-IX, exosome markers and intraluminal low pH co-localize in exosome released by prostate cancer cells in acidic conditions, as shown by confocal microscopy. A significant difference in CA IX expression was that circulating exosomes of cancer patients expressed a double band at 58/54 kDa, suggesting the occurrence of post-transcriptional changes of CA IX expression induced by the tumour condition.

All in all our study shows for the first time a correlation between CA IX expression and intraluminal acidity in plasmatic exosomes of cancer patients. Confocal microscopy showed that a high intracellular acidity characterises prostate cancer cells stably cultured at pH 6.5, as demonstrated by the massive intake of BCECF in these cells; this was consistent with a clear co-localization of the exosomal marker CD9 and CA IX, mainly at the plasma membrane, while co-expressed at the cytoplasm and the nuclear membrane levels, as well. The concentration of CA IX/CD9/acidic particles at the plasma membrane suggests an increased activity of the endosomal compartment, in turn, leading to exosome formation and extracellular release. These results indicate that tumour acidic microenvironment is the crucial factor in affecting the CA IX expression and activity in prostate cancer cells and cancer-released exosomes. It appears conceivable that the microenvironmental tumour acidity may increase exosomes release, together with the upregulation of CA IX expression and activity in PCa. Therefore, the increase of plasmatic levels of CA IX/acidic exosomes in prostate cancer patients may be the result of the spill-over of these exosomes from the tumour to the bloodstream. Indeed CA IX is a transmembrane protein whose catalytic domain exhibits the fundamental biochemical and biophysical properties that allow stability and activity at low pH^34^^,^^50^. Conversely, CA IX expression and activity is down-regulated in healthy donors plasma exosomes since under normal conditions, the cells don’t need a high expression and activity of the proton exchangers because they use oxidative metabolism. The key role of the acidic tumour microenvironment in increasing the release of exosome by cancer cells support our previous studies^16^^,^^17^^,^^27^^,^^30^.

Our study supports a new idea for providing reliable tumour markers suitable for clinical use. Here, we have shown that plasmatic exosomes from cancer patients overexpress CA IX and together with exerting a CA IX-related activity. This is probably the first translational evidence in this sense; that is the expression of a protein plus the activity related to the expressed protein, may represent a new and non-invasive diagnostic tool with a potentially high clinical impact in early diagnosis and monitoring of prostatic cancer, but conceivably all malignant tumours as well.
